# Exosomal miR-24-3p mediates myoblast-macrophage crosstalk to promote abdominal muscle repair

**DOI:** 10.3389/fphar.2025.1604776

**Published:** 2025-06-03

**Authors:** Yuchen Liu, Zhenyu Zou, Jinxin Cao, Tong Zhu, Yilin Zhu, Yingmo Shen

**Affiliations:** Department of Hernia and Abdominal Wall Surgery, Beijing Chaoyang Hospital, Capital Medical University, Beijing, China

**Keywords:** exosomal miR-24-3p, myoblast, macrophage, abdominal muscle repair, muscle regeneration

## Abstract

**Objective:**

The objective of this study was to explore the role of exosomal miR-24-3p in facilitating communication between myoblasts and macrophages, and to assess its potential in promoting abdominal muscle repair.

**Methods:**

We utilized C2C12 myoblasts and RAW 264.7 macrophages, inducing the latter into an M2 phenotype. miR-24-3p levels were manipulated via transfection, and exosomes were isolated from M2 macrophages using ultracentrifugation. Exosome characterization was performed using TEM and Western blot. *In vitro* assays evaluated C2C12 cell proliferation, migration, and differentiation. *In vivo*, a cardiotoxin-induced mouse model of muscle injury was used to assess the effects of exosomal miR-24-3p on muscle repair, including histological assessment and analysis of cytokine and metabolic markers.

**Results:**

Our results demonstrated that exosomal miR-24-3p, when isolated from M2 macrophages, was effectively internalized by C2C12 cells and significantly enhanced their metabolic activity, proliferation, and migratory capabilities. Moreover, it induced cellular differentiation, as observed under microscopic examination. In the abdominal muscle injury model, the administration of exosomal miR-24-3p led to a reduction in muscle fiber damage, fibrosis, and inflammation. It also promoted the restoration of glucose and lipid metabolism, which is critical for the energy demands of regenerating muscle. Furthermore, exosomal miR-24-3p upregulated the expression of genes associated with muscle cell proliferation and differentiation, suggesting its potential role in muscle repair.

**Conclusion:**

In conclusion, exosomal miR-24-3p plays a significant role in facilitating abdominal muscle repair by mediating the interaction between myoblasts and macrophages.

## Graphical Abstract

**Figure:**
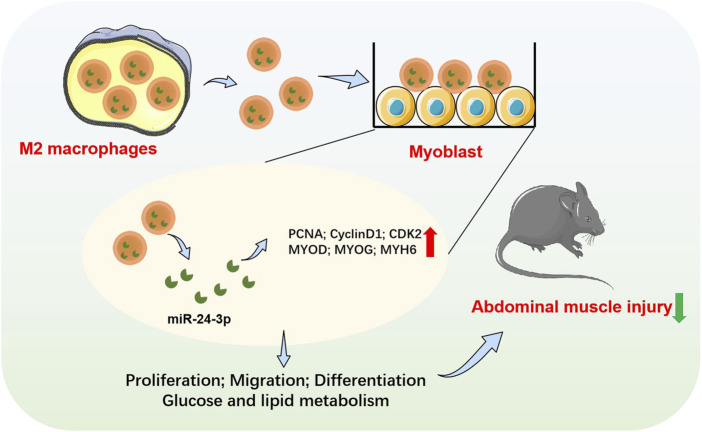
Schematic illustration of exosomal miR-24-3p-mediated crosstalk between M2 macrophages and C2C12 myoblasts, promoting abdominal muscle repair via enhanced proliferation, differentiation, and metabolic regulation.

## 1 Introduction

Abdominal muscle injuries, involving the rectus abdominis, oblique muscles, transverse abdominis, and associated musculature, are structural damage caused by external forces, physical activities, or other etiologies (Giarda et al., 2024). These injuries are commonly associated with pain, swelling, and functional deficits, with severe instances potentially disrupting daily activities and athletic capabilities (Wiik Larsen et al., 2022). A survey revealed that only 449 out of 7,202 trauma patients (6.2%) experienced abdominal muscle injuries (Wiik Larsen et al., 2022). Most of these injuries can be mitigated through rest, physical therapy, and pharmacological interventions. Physical therapy often includes muscle-strengthening and stretching exercises to facilitate functional recovery and preempt future injuries. Severe abdominal muscle injuries, especially those with muscle tears or ruptures, may necessitate surgical intervention. Following surgery, patients typically undergo rehabilitation to restore abdominal strength and function (Giarda et al., 2024). Although infrequent in sports, abdominal muscle injuries significantly affect athletic performance and quality of life. Hence, a profound comprehension of the muscle repair mechanisms is imperative for reducing the prevalence of such injuries.

The complex muscle repair process is characterized by the intricate interplay of diverse cellular and molecular elements, with myoblasts and macrophages assuming critical roles. Myoblasts, synonymous with satellite cells, serve as the principal muscle precursors, playing an indispensable part in the genesis of new muscle fibers. Upon muscle injury, these cells are activated, proliferate, and subsequently differentiate into myotubes, ultimately fusing with the damaged muscle fibers to generate new, viable muscle fibers (Gabellini, 2022; Olenic et al., 2024). Macrophages are equally instrumental in muscle regeneration, as they eliminate inflammatory responses and debris, thereby creating a supportive milieu for muscle cell reconstitution (Rybalko et al., 2015). In addition, macrophages release an array of growth factors, including fibroblast growth factor and hepatocyte growth factor, which stimulate the proliferation and differentiation of muscle cells (Rybalko et al., 2015). Research has underscored the importance of the intercellular communication between myoblasts and macrophages for efficient muscle repair post-injury (Fang et al., 2021; Rodríguez et al., 2024; Rybalko et al., 2015). Through their varied phenotypes and the secretion of a multitude of factors, macrophages foster a propitious setting for myoblasts, thus facilitating muscle regeneration and repair. This sophisticated interaction is paramount for the efficacy of muscle repair and the restoration of function (Fang et al., 2021; Rodríguez et al., 2024; Rybalko et al., 2015). A more profound comprehension of these mechanisms is not only instrumental in deciphering the post-injury repair dynamics but also lays a crucial theoretical groundwork for the formulation of novel therapeutic approaches.

MicroRNAs (miRNAs), a class of small non-coding RNA molecules, exert post-transcriptional regulation of gene expression and are implicated in diverse biological processes, including cell proliferation, differentiation, and inflammation (Leitão and Enguita, 2022). While miRNAs are well-known for their roles in cancer biology (Yuan et al., 2014), their significance in muscle repair has increasingly gained attention. Within the realm of muscle biology, miRNAs have been demonstrated to modulate muscle cell function, with their significance in muscle repair garnering escalating interest. Empirical studies have illustrated that the local administration of miR-1, miR-133, and miR-206 in a rat model of skeletal muscle injury can significantly enhance muscle regeneration both morphologically and physiologically, while also effectively mitigating fibrosis (Nakasa et al., 2010).

Extracellular vesicles, particularly exosomes, have attracted considerable interest due to their cellular trafficking capabilities. These small extracellular vesicles, measuring 30–150 nm in diameter and encased in a lipid bilayer, are secreted by a diverse array of cell types, including macrophages (Miron and Zhang, 2024). Exosomes serve as conduits for intercellular communication, equipped with the capacity to convey bioactive molecules—including miRNAs, proteins, and lipids—to both distant and proximate cells, thereby altering the biology of the recipient cells (Jadamba et al., 2024). Recent research has elucidated the pivotal role of exosomes in the repair of muscle injuries (Wan R. et al., 2024). Exosomes initiate the activation of muscle stem cells and facilitate their differentiation into new muscle fibers, which expedites the muscle regeneration process. Notably, in the early stages of skeletal muscle injury, fibro-adipogenic progenitor cells secrete exosomes rich in miR-127-3p, which are instrumental in the activation and differentiation of muscle stem cells (Yu et al., 2024). Moreover, these exosomes enhance the *in vitro* myogenesis of C2C12 myoblasts and the angiogenesis of HUVECs, ensuring the necessary blood supply to the newly formed muscle tissue (He et al., 2022). They also accelerate *in vivo* skeletal muscle regeneration in a cardiotoxin-induced muscle injury model (Singla et al., 2019). Exosomes mitigate fibrosis and inflammation post-muscle injury by carrying specific microRNAs, including miR-21, miR-23a, miR-125b, and miR-145, which inhibit the TGF-β2/SMAD2 signaling pathway and suppress the formation of myofibroblasts, thus preventing scar tissue formation (Fang et al., 2016). Their anti-inflammatory effects are also crucial in reducing inflammatory responses, which is essential for muscle repair and functional recovery (Wan et al., 2022). Furthermore, exosomes elevate muscle function by activating the expression of genes associated with muscle generation, such as MYOG, MYOD, myogenin, Pax7, and eMyhc (Wan et al., 2022). Consequently, exosomes hold the potential to serve as therapeutic agents for muscle repair.

In light of these findings, our study aims to investigate the role of exosomal miR-24-3p in mediating the crosstalk between myoblasts and macrophages, and its potential to promote abdominal muscle repair. Our results provide novel insights into the molecular mechanisms underlying muscle regeneration and may pave the way for developing targeted therapeutic strategies to enhance muscle healing.

## 2 Materials and methods

### 2.1 Cell culture

C2C12 myoblasts and the RAW 264.7 murine macrophage cell line (ATCC®TIB-71, Manassas, Virginia, USA) were cultured in Dulbecco’s modified Eagle’s medium (DMEM) (glucose 25 mM) containing 10% serum (10099141C, Gibco, USA), and 1% dual antibody (penicillin/streptomycin) (10099141C, Gibco, USA), all at an incubation temperature of 37°C in an atmosphere of 5% CO_2_. The culture medium was refreshed every 2 days. When the RAW 264.7 cells grow to 80%–90%, passaging was performed. The above reagents were purchased from Thermo Fisher Scientific (Waltham, USA). For mouse C2C12 myoblasts, when the cell density reached 70%, the cells were trypsinized using 0.25% trypsin-EDTA, and then re-plated into new culture dishes. Upon reaching a density of 90%, the C2C12 cells were switched to DMEM differentiation medium (DM) containing 2% horse serum for myogenic induction and differentiation.

### 2.2 Directed induction into M2-like macrophages

To induce the generation of M2-like macrophages, macrophages were stimulated with IL-4 and IL-10 at a concentration of 10 ng/mL for a period of 7 days. The quantification of these M2-type macrophages was achieved using flow cytometry (BD FACSCalibur, BD Biosciences, San Jose, CA, USA). And data were analyzed with FlowJo software (version 10.8, FlowJo LLC, Ashland, OR, USA). Specifically, M2-polarized macrophages were characterized by the expression profile of CD11b^+^ CD68^+^ CD11c^−^/CD206^+^.

### 2.3 Cells transfection

Based on the sequence of miR-24a-3p, corresponding inhibitor sequences were designed to construct the RNA oligonucleotides. On the day prior to transfection, C2C12 cells were seeded into six-well plates. In Eppendorf tube number 1: 125 μL of Opti-MEM medium plus 5 μL of Lipofectamine 3000; in Eppendorf tube number 2: 125 μL of Opti-MEM medium plus 5 μL of miRNA. The contents of tube number 2 were added to tube number 1 to form miRNA-lipid complexes, which were thoroughly mixed and incubated at room temperature for 15 min. The miRNA-lipid complexes were then added to the cells in the six-well plates. After 6 h, the medium was replaced, and the cells were cultured for 24–48 h for subsequent experiments. Transfection efficiency was verified through antibiotic selection and qRT-PCR.

### 2.4 Exosome isolation

Exosomes were extracted from the conditioned medium of M2-like macrophages using a differential ultracentrifugation method. The process began with sequential centrifugation steps at 300 × g, 2,000 × g, and 10,000 × g (all at 4°C), with supernatants collected after each spin. Afterward, the supernatant was subjected to ultracentrifugation at 100,000 × g (4°C), and the pellet obtained was collected for further analysis. The pellet containing exosomes was resuspended in pre-filtered PBS (pH 7.4, 0.2 μm) for subsequent TEM analysis.

### 2.5 Exosome identification

To characterize exosomes, the purity and morphology of exosomes were assessed using transmission electron microscopy (TEM, Hitachi H7500 TEM, Tokyo, Japan). Positive exosomal markers (ALIX, CD63, and TSG101) in exosomes were analyzed using Western blotting.

### 2.6 TEM assay

M2-exo solution, in a 10 μL volume, was applied to carbon-coated copper grids. The samples were left to air-dry for 5 min before being stained with 1% uranyl acetate (Sigma-Aldrich) for another 5 min. Post-staining, the grids were air-dried for an additional 20 min prior to examination with an 80 kV transmission electron microscope (Hitachi H7500 TEM, Tokyo, Japan).

### 2.7 Exosome labeling and Co-culture

Exosomes were labeled with fluorescence using the PKH 26 Green Fluorescent Cell Ligand Mini Kit (Sigma-Aldrich, MINI26). Following a 5-minute incubation at room temperature, the labeling reaction was stopped by adding an equal volume of exosome-free fetal bovine serum. The exosomes were then washed twice with PBS to eliminate any unbound dye, after which confocal microscopy (Olympus FV1000, Tokyo, Japan) was used to capture images. C2C12 cell nuclei were stained with DAPI for 30 min and subsequently washed twice with PBS. Subsequently, the exosomes and cells were co-cultured, and the fluorescent outcomes were visualized using confocal microscopy.

### 2.8 CCK-8

C2C12 cells were digested enzymatically, centrifuged, and then seeded into 96-well plates for standard cultivation. Subsequently, 10 μL of CCK-8 solution (Beyotime, Shanghai, China) was introduced into each well at the designated time. The plates were incubated at 37°C for 2 h in a regulated setting. Following this, the optical density at 450 nm (OD450) was determined using an enzyme-linked immunosorbent assay (ELISA) reader (Molecular Devices, SpectraMax i3x, Sunnyvale, CA, USA).

### 2.9 EdU

To evaluate cell proliferation, we employed the Cell-Light EdU DNA Cell Proliferation Kit (Ribobio, Guangzhou, China). C2C12 cells were plated at a concentration of 2.5 × 10^4^ cells per well in 96-well plates and exposed to EdU for 2 h at a final concentration of 50 μM. After fixation with 4% paraformaldehyde for 30 min and permeabilization with 0.5% Triton X-100 for 10 min, the cells were treated with Apollo solution (1x, 30 min) and stained with DAPI. Post-staining, the cells were examined under fluorescence microscopy. The count of positive cells was calculated as a percentage of the total cell count.

### 2.10 Wound healing assay

C2C12 cells were evenly seeded in 6-well plates and maintained in FBS-supplemented medium until they achieved 90% confluence. A sterile pipette tip was then used to make a single straight scratch through the cell layer. After that, the plates were filled with serum-free medium, and the cells were cultured for an additional 24 h. The migration of the cells was documented with an Olympus CKX41 inverted microscope (Tokyo, Japan) and analyzed using ImageJ software (version 1.53k, NIH, Bethesda, MD, USA). The migration rate (MR) was calculated using the formula: MR = (A− B)/A × 100%, with A being the initial scratch width at time zero and B being the width remaining after 24 h of incubation.

### 2.11 qRT-PCR

RNA was extracted from C2C12 cells and mouse abdominal tissues using Trizol reagent (Takara, Tokyo, Japan). Following RNA quantification, cDNA synthesis was conducted using 500 ng of RNA with the GoScript RT System from Promega (Charbonnières, France). Subsequently, quantitative PCR (qPCR) was performed using iQ™ SYBR^®^ Green Supermix (Bio-Rad, München, Germany) along with specific primer sets. The relative expression levels, as determined from the qPCR data, were calculated using the 2^−ΔΔCt^ method and are expressed as mean fold changes with the standard error of the mean (SEM). For normalization purposes, GAPDH served as a housekeeping gene. Primer sequences are listed in Table 1.

**TABLE 1:** Primers for qRT-PCR.

Gene	Forward (5′–3′)	Reverse (5′–3′)
GAPDH	ACC​ACA​GTC​CAT​GCC​ATC​AC	TCC​ACC​ACC​CTG​TTG​CTG​TA
PCNA	TCC​AGA​ACA​AGA​GTA​TAG​C	TACAACAGCATCTCCAAT
CyclinD1	CCGTCCATGCGGAAGATC	CAG​GAA​GCG​GTC​CAG​GTA​G
CDK2	TTT​GCT​GAG​ATG​GTG​ACC​CG	TAA​CTC​CTG​GCC​AAA​CCA​CC
MyoD	ACG​GCA​TGA​TGG​ACT​ACA​GC	AGG​CAG​TCG​AGG​CTC​GAC​A
MyoG	CAAATCCACTCCCTGAAA	GCA​TAG​GAA​GAG​ATG​AAC​A
MYPT1	AGT​TAA​TCG​GCA​AGG​GGT​TGA	ATG​ACC​ACT​ATT​TAG​CCA​CTG​C
VEGFR-1	GAA​TTA​TTT​TAG​GAC​CAG​GA	AAA​CTC​CCA​CTT​GCT​GGC​AT
MYH6	GCA​ACA​TGG​AGG​GCG​AGA​TA	CAC​TTT​GCA​TTC​ACC​GCC​TC

### 2.12 Western blot

Tissue or cell samples were collected and lysed in RIPA buffer for a duration of 30 min. Post-lysis, centrifugation was performed at 12,000 × g for 15 min at 4°C, resulting in the collection of supernatants. The BCA assay kit was utilized to measure protein concentrations, ensuring equivalent loading of proteins. SDS-PAGE was set up for protein separation via electrophoresis. Subsequently, proteins were transferred to PVDF membranes, employing either semi-dry or wet transfer methods. The membranes were blocked with 5% BSA or skim milk for 2 h to reduce non-specific binding. Primary antibodies, including anti-PGAM5 (1:5000), were incubated overnight at 4°C. The next day, the membranes underwent three washes. Then, HRP-conjugated anti-rabbit or anti-mouse secondary antibodies (1:3000) were applied and incubated for 2 h at room temperature. After further washing, the bands were developed using detection reagents. All antibodies used in this study, including anti-GLUT4, anti-AKT2, anti-G6PC, anti-GYS1, anti-PLIN4, anti-FABP4, anti-FASN and anti-GAPDH, were purchased from Cell Signaling Technology (Danvers,MA,USA).

### 2.13 Animal model and groups

A total of 24 male C57BL/6 mice (6 weeks of age), were procured from Beijing Huakang Biotechnology Co., Ltd. Prior to the commencement of the experiment, these mice were housed in an environment maintained at a temperature of 20°C–26°C and a humidity level of 40%–70%, under a regimen of 12 h of alternating light and darkness. During this 7-day acclimatization period, the rats had access to ample water and food.

Within a biosafety cabinet, the lyophilized powder of cardiotoxin (CTX) was diluted to a concentration of 10 μM using sterile PBS, aliquoted, and stored at −80°C to prevent repeated freeze-thaw cycles. Mice were anesthetized with isoflurane, and 100 μL of CTX was administered in three separate injections directly into the abdominal muscle tissue using an insulin syringe. The control group received an equivalent volume of 10% glucose solution. After 7 days, the mice were euthanized, and their abdominal muscle tissues were collected.

In accordance with experimental requirements, the model mice were categorized into the following groups: Abdominal Muscle Injury group (AM); Abdominal Muscle Injury + Exosome Control group (AM-Exo-miR-NC); and Abdominal Muscle Injury + Exosome group (AM-Exo-miR-inhibitor). The Abdominal Muscle Injury group (AM) underwent muscle injury treatment only. Building upon this, the AM-Exo-miR-NC group was injected with exosomes derived from M2 macrophages transfected with miR-NC at a concentration of 2 × 10^9^. Similarly, the AM-Exo-miR-inhibitor group received exosomes from M2 macrophages transfected with miR-inhibitor, also at a concentration of 2 × 10^9^.

### 2.14 Histology

Prior to euthanasia, mice were intraperitoneally injected with a 1% Evans Blue solution. If the mice’s sarcolemma was compromised, the dye would penetrate into the cells, staining them blue, thereby indicating the extent of muscle damage. After euthanizing the experimental mice, abdominal muscle tissues were harvested and fixed in 4% paraformaldehyde for 48 h. The tissues were then dehydrated, cleared, and embedded in paraffin. Slides and cover slips were soaked overnight in nitric acid, followed by sequential rinsing with tap water and distilled water, and wiped with anhydrous alcohol before being placed in a clean, dust-free area. The slides were treated with poly-L-lysine adhesive for subsequent use. Paraffin-embedded blocks were sectioned into 5 μm consecutive slices using standard procedures. The sections were deparaffinized with two changes of xylene for 10 min each, followed by a 5-minute soak in 50% xylene. They were then rinsed twice in anhydrous ethanol for 5 min each, and sequentially soaked in 95%, 80%, and 70% ethanol for 5 min each, followed by three rinses in PBS and distilled water for 2 min each. The prepared sections were subjected to H&E staining and Masson’s trichrome staining to assess the pathological structural changes and collagen deposition in the abdominal muscle tissues. Additionally, frozen sections of the abdominal muscle tissues were stained with Oil Red O to observe lipid deposition.

### 2.15 ELISA

Utilizing the Enzyme-linked immunosorbent assay (ELISA) kit from Hangzhou Lianke Biotechnology Co., LTD in Hangzhou, China, we assessed the levels of pro-inflammatory cytokines, including TNF-α and IL-6, in and mouse serum, following the kit’s provided guidelines.

### 2.16 Luciferase reporter assay

For luciferase reporter assays, the wild-type (WT) and mutant (MUT) 3′-UTR sequences of Notch containing the predicted miR-24-3p binding site were cloned into a luciferase reporter vector. Myoblasts were co - transfected with miR-24-3p mimic or miR-NC mimic and the corresponding reporter vector. After 48 h, luciferase activity was measured using a luciferase assay kit according to the manufacturer’s instructions.

### 2.17 Statistical analysis

All results are depicted as the mean with standard error of the mean (SEM). When comparing the two groups, a Student’s t-test was utilized. To evaluate the statistical significance of differences across multiple groups, a one-way ANOVA was conducted, supplemented by Tukey’s *post hoc* analysis. The statistical computations were performed using GraphPad Prism software (San Diego, CA, USA), and a P value of less than 0.05 was set as the threshold for statistical significance.

## 3 Results

### 3.1 Preparation and characterization of M2-exos

**FIGURE 1:**
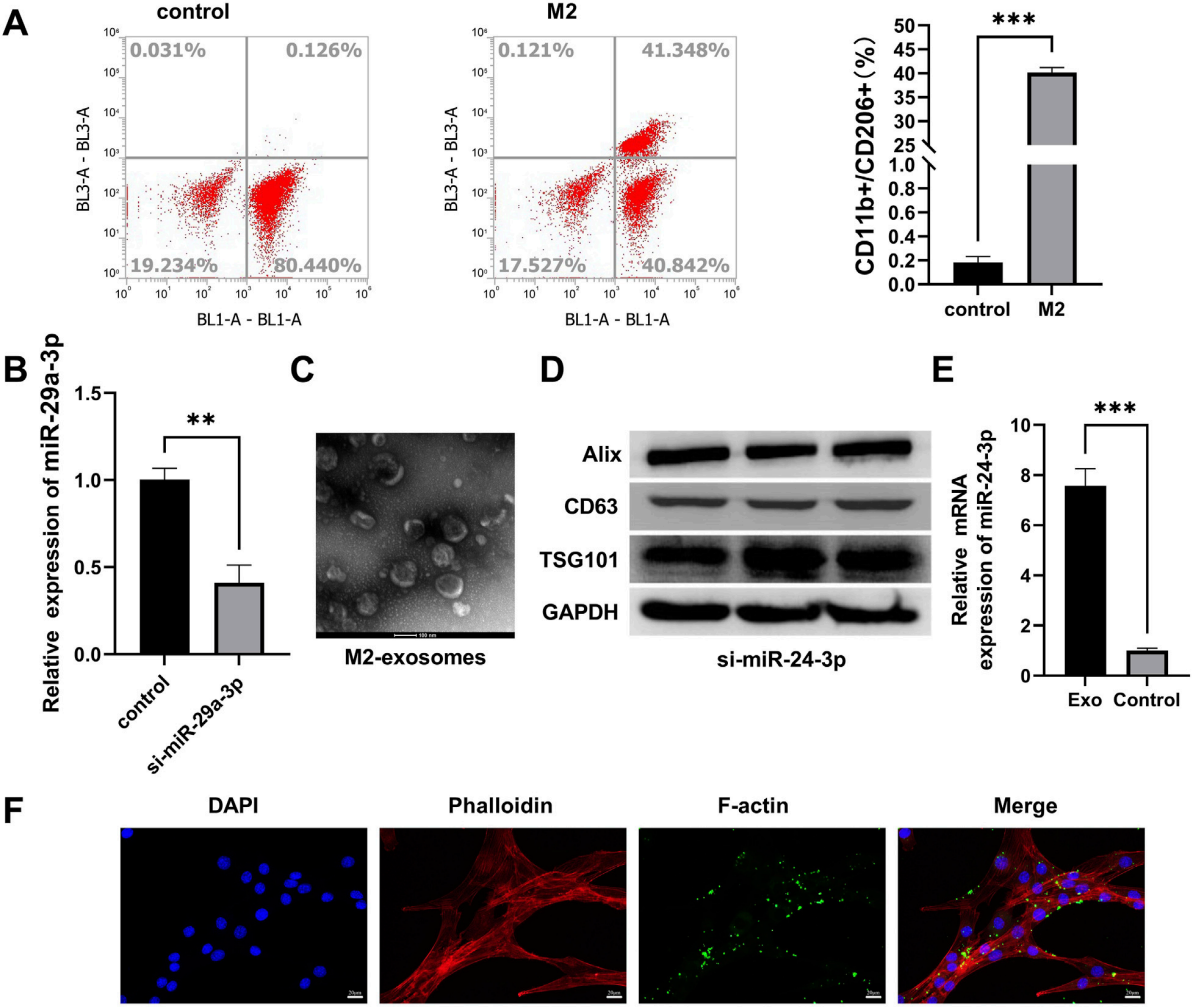
Preparation and characterization of M2 macrophage-derived exosomes (M2-exos). **(A)** Flow cytometry analysis of RAW 264.7 macrophages induced with IL-4/IL-10 (10 ng/mL, 7 days) for M2 polarization, gated on CD11b^+^ CD68^+^ CD11c^−^/CD206^+^ populations. **(B)** Validation of miR-24-3p inhibitor transfection efficiency in M2 macrophages via qRT-PCR. **(C)** Transmission electron microscopy (TEM) imaging of isolated exosomes (scale bar: 100 nm). **(D)** Western blot analysis of exosomal markers (ALIX, CD63, TSG101) in M2-exos. **(E)** mRNA levels of miR-24-3p were analyzed using quantitative RT-PCR. **(F)** Confocal microscopy imaging of PKH26-labeled M2-exos (red) internalized by DAPI-stained C2C12 myoblasts (blue; scale bar: 20 μm). n = 3, ***p < 0.001.

To induce M2 polarization in RAW264.7 macrophages, RAW264.7 cells were incubated with IL-4 and IL-10 for 7 days. Flow cytometry analysis revealed an upregulation in the proportion of CD11b^+^ CD68^+^ CD11c^−^/CD206^+^ cells, indicating successful M2 polarization (Figure 1A). M2 macrophages with downregulated miR-24-3p expression were obtained through transfection and validated for transfection efficiency by qRT-PCR after antibiotic selection. Compared to the control group, the expression level of miR-24a-3p in M2 macrophages transfected with miR-24a-3p inhibitors was significantly reduced (Figure 1B). M2 macrophages were cultured and the supernatant was collected. M2-exosomes were isolated from the supernatant by differential centrifugation. As shown in Figure 1C, the morphology and structure of M2-exosomes were examined using TEM. The results indicated that these exosomes were either hemispherical or disc-shaped with a concave side and particle sizes ranging from 50 to 150 nm. Expression analysis of exosomal marker proteins, including Alix, CD63, and TSG101, showed an increase in Alix expression in M2-polarized macrophage-derived exosomes, a decrease in CD63 expression, and no significant change in TSG101 expression (Figure 1D). By performing qRT-PCR, we found that the relative expression of miR-24-3p in exosomes was significantly increased in the experimental group compared to the control group, as shown in the new Figure 1E. Furthermore, M2 macrophage-derived exosomes, labeled with PKH67, were co-cultured with C2C12 cells. Fluorescence staining demonstrated the internalization of exosomes by C2C12 cells (Figure 1F). These experimental results confirm the successful isolation of M2 macrophage-derived exosomes and their uptake by C2C12 cells. Notably, while ALIX (a core exosomal marker) was upregulated, CD63 (a commonly reported exosomal tetraspanin) showed reduced expression in M2-exosomes. This unusual pattern may reflect M2 macrophage-specific exosomal biogenesis or miR-24-3p-dependent modulation of marker protein levels, warranting further investigation in future studies to clarify the underlying mechanisms.

### 3.2 Exosomal miR-24-3p enhances myoblast proliferation and migration

**FIGURE 2:**
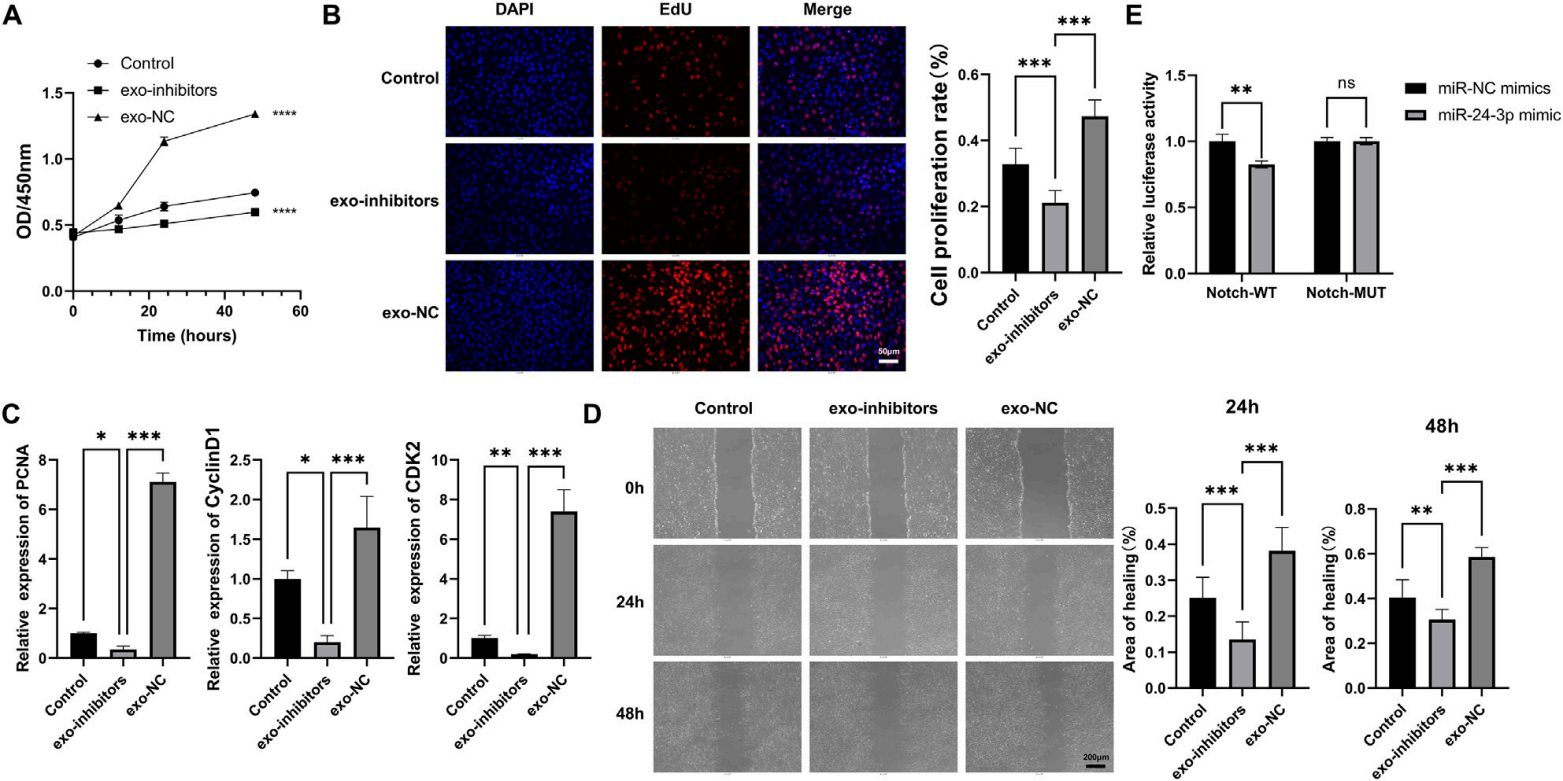
Effects of M2-exosomal miR-24-3p on myoblast proliferation and migration. **(A)** CCK-8 assay to assess metabolic activity of C2C12 cells treated with PBS, exo-NC (control exosomes), or Exo-inhibitor (miR-24-3p-suppressed exosomes). **(B)** EdU staining (red) for quantification of proliferating C2C12 cells (DAPI counterstain, blue; scale bar: 50 μm). **(C)** qRT-PCR analysis of proliferation-related genes (PCNA, Cyclin D1, CDK2) in C2C12 cells. **(D)** Wound healing assay to evaluate C2C12 cell migration post-treatment (images captured at 0 h and 24 h; scale bar: 200 μm). **(E)** Luciferase reporter assays using Notch wild-type (Notch-WT) and mutant (Notch-MUT) reporter vectors co-transfected with miR-24-3p mimic or miR-NC mimic. n = 3, *p < 0.05, **p < 0.01, ***p < 0.001.

In order to elucidate the effects of exosomal miR-24-3p on myoblasts, we performed co-culture experiments with PBS, exo-NC, exo-inhibitors, and C2C12 cells. The CCK-8 assay demonstrated that exo-NC significantly enhanced the metabolic activity and vitality of C2C12 cells compared to the PBS group, while co-culturing with exo-inhibitors led to a marked decrease in cellular activity (Figure 2A). The impact of exosomal miR-24-3p on cellular proliferation was assessed using EdU incorporation, which revealed that the addition of exo-NC significantly boosted the proliferative capacity of C2C12 cells, whereas the addition of exo-inhibitors resulted in an opposing effect (Figure 2B). Subsequently, we utilized qRT-PCR to examine the expression of key proliferation factors in myoblasts, including PCNA, CyclinD1, and CDK2. Consistent with our expectations, exo-NC elevated the mRNA levels of these factors in C2C12 cells, while exo-inhibitors suppressed their expression (Figure 2C). Furthermore, wound healing assays indicated that cells co-cultured with exo-NC exhibited enhanced migratory ability compared to the Control group, whereas the migration rate of cells in the exo-inhibitors group was significantly slower, followed by the M1-exos group (Figure 2D). Collectively, these findings confirm that exosomal miR-24-3p can induce proliferation and migration in C2C12 cells.

To validate Notch as a downstream target of miR-24-3p, luciferase reporter assays were conducted. As shown in Figure 2E, in cells transfected with Notch-WT reporter vector, the relative luciferase activity was significantly reduced (*P* < 0.01) when co-transfected with miR-24-3p mimic compared to miR-NC mimic. However, in cells with Notch-MUT reporter vector, there was no significant difference in luciferase activity between miR-24-3p mimic and miR-NC mimic groups. These results demonstrate a direct binding of miR-24-3p to the 3′-UTR of Notch, validating Notch as a downstream target of miR-24-3p in myoblasts.

### 3.3 Exosomal miR-24-3p induces myoblast differentiation

**FIGURE 3:**
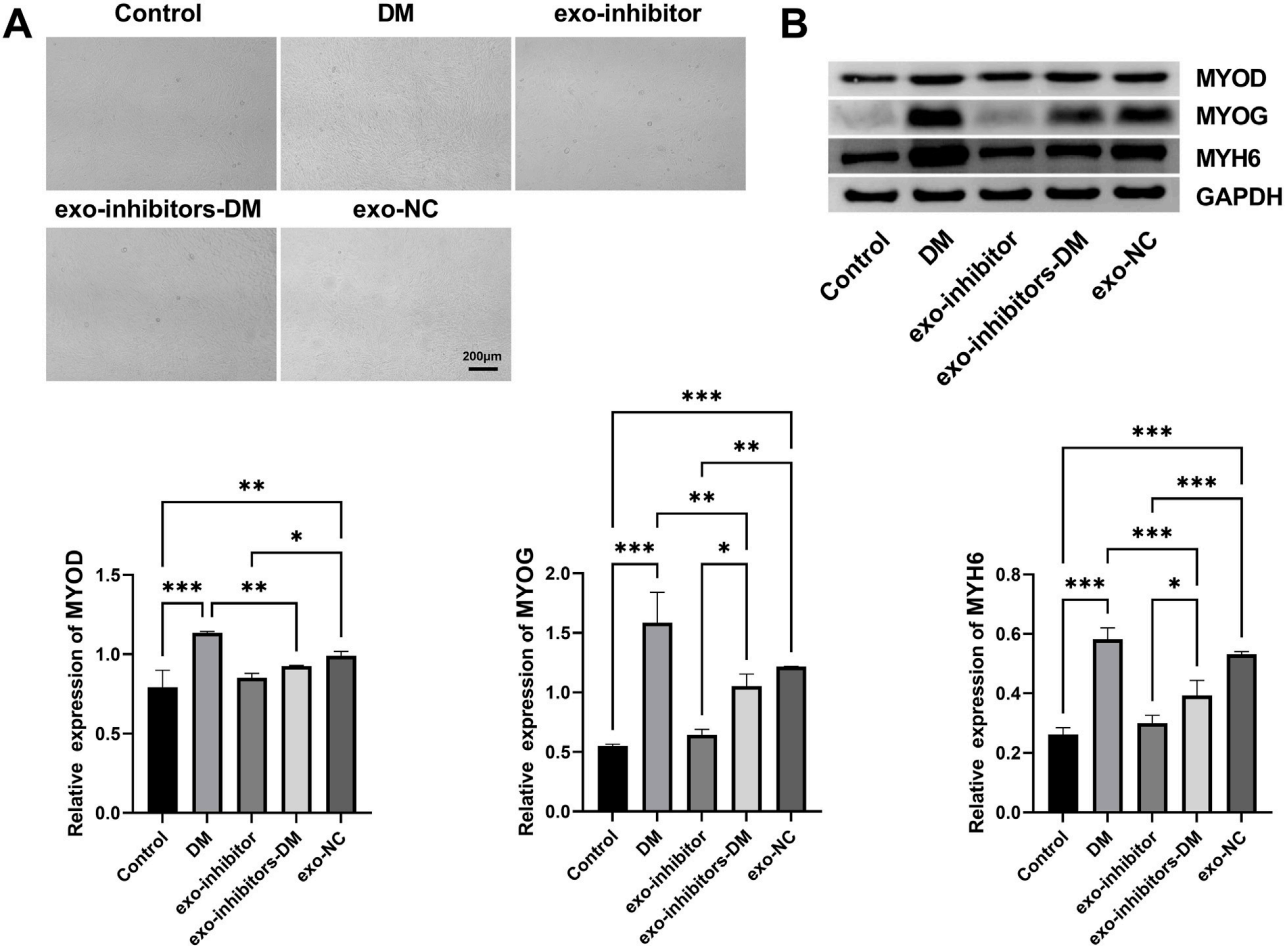
Exosomal miR-24-3p induces myoblast differentiation. **(A)** Microscopic evaluation of C2C12 myoblast differentiation under indicated experimental conditions (Control, DM, exo-inhibitors-DM, exo-NC) (scale bar: 200 μm). **(B)** Western blot analysis of myogenic markers MYOD, MYOG, and MYH6 in C2C12 cells following experimental treatments. Loading control: GAPDH. n = 3, *p < 0.05, **p < 0.01, ***p < 0.001.

To ascertain the role of exosomal miR-24-3p in C2C12 cell differentiation, exo-NC and exo-inhibitors were transfected into C2C12 cells, followed by myogenic induction. Observations and photographic documentation were conducted at days 2 (D2), 4 (D4), and 6 (D6) post-differentiation using a microscope. As shown in Figure 3A, C2C12 cells in the Control group exhibited a low degree of differentiation; whereas, the DM group demonstrated distinct differentiation characteristics, with the formation of visible myotubes under the microscope, indicating effective induction of the differentiation process. The differentiation degree of C2C12 cells in the Exo-inhibitor and exo-NC groups was similar to the Control group, with fewer myotubes observed under the microscope; in the exo-inhibitors-DM group, the presence of exosomal inhibitors seemed to counteract the differentiation-promoting effect of the differentiation medium, resulting in a noticeable reduction in myotube formation compared to the DM group. Further analysis was conducted using Western blot to assess the expression of key proteins involved in myocyte differentiation, including MYOD, MYOG, and MYH6, across all groups. Similar to the Control group, the protein expression levels of MYOD, MYOG, and MYH6 in the Exo-inhibitor and exo-NC groups did not significantly change, suggesting a limited impact of exosomal inhibitors on myocyte differentiation. Upon induction with the differentiation medium, the content of these proteins in C2C12 cells was markedly elevated, aligning with the microscopic observations. Notably, although the protein expression levels of MYOD, MYOG, and MYH6 in the exo-inhibitors-DM group increased, the increase was significantly less than that in the DM group alone (Figure 3B). These findings indicate that the DM can effectively promote myocyte differentiation, while the presence of Exo-inhibitor may inhibit this process.

### 3.4 Exosomal miR-24-3p ameliorates abdominal muscle injury in C57BL/6J male mice

**FIGURE 4:**
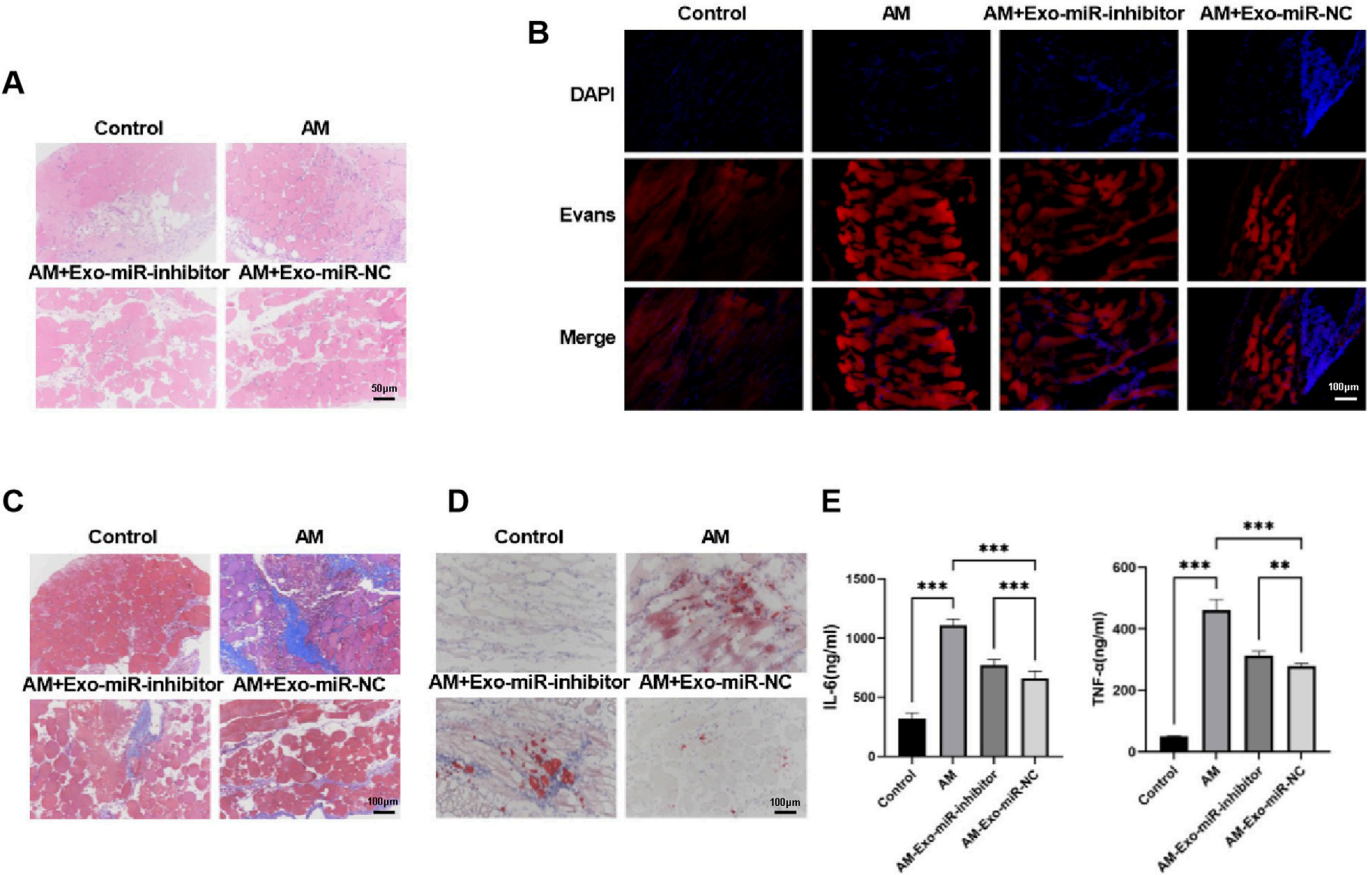
Exosomal miR-24-3p ameliorates abdominal muscle injury in C57BL/6J male mice. **(A)** Hematoxylin and eosin (HE) staining was performed on paraffin-embedded sections of injured abdominal muscle tissues to evaluate histological architecture and inflammatory cell infiltration (scale bar: 50 μm). **(B)** Evans Blue dye uptake assay was conducted to assess muscle fiber integrity and regeneration status at the injury site (scale bar: 100 μm). **(C)** Masson’s trichrome staining was employed to quantify collagen deposition and fibrosis in regenerating abdominal muscle tissues (scale bar: 100 μm). **(D)** Oil Red O staining of cryosectioned muscle tissues was used to visualize lipid accumulation and metabolic alterations in the injured region (scale bar: 100 μm). **(E)** Enzyme-linked immunosorbent assay (ELISA) measured serum levels of pro-inflammatory cytokines (TNF-α, IL-6) in experimental and control groups. n = 3, **p < 0.01, ***p < 0.001.

Building upon our preliminary findings, we further utilized C57BL/6J male mice to establish a muscle injury model and assessed the impact of exosomal miR-24-3p on muscle injury repair at the animal level. Following the induction of muscle injury with cardiotoxin, we performed hematoxylin and eosin (H&E) staining on abdominal tissues from each group to understand the basic morphological changes in the muscles (Figure 4A). The Control group mice exhibited a regular polygonal state in muscle morphology and appearance, with muscle fiber nuclei located peripherally within the cells, tight connections between muscle fibers, and no discernible gaps, indicating a well-organized structure. The intermuscular bundles were evenly spaced, with visible microvessels and interstitial cells, and no signs of fat infiltration or fibrosis were observed. The Abdominal Muscle (AM) group showed destruction of muscle fiber tissue architecture, with a significant aggregation of inflammatory cells within the muscle fibers and nuclei deformation into a rod-like shape, confirming the successful establishment of the muscle injury model. In contrast, the AM-Exo-miR-NC group showed a marked reduction in muscle fiber damage and inflammation, suggesting a reparative effect of exosomal miR-24-3p. Although the AM-Exo-miR-inhibitor group also demonstrated improvements in symptoms, the effects were significantly weaker compared to the AM-Exo-miR-NC group.

Evans Blue staining revealed a higher degree of staining in the Abdominal Muscle (AM) group compared to the Control group, indicating severe muscle damage. However, a significant reduction in staining was observed in the AM-Exo-miR-NC group, suggesting that exosomal miR-24-3p can ameliorate muscle injury, while the Exo-miR-inhibitor diminished the therapeutic effect of miR-24-3p (Figure 4B). Subsequently, Masson’s staining was employed to assess collagen deposition in the abdominal muscle tissues. As shown in Figure 4C, the Control group displayed well-preserved muscle fiber morphology with regular polyhedral shapes, tightly apposed muscle fibers, and clearly visible intermuscular bundles with orderly spacing, including interstitial elements such as faintly blue microvessels; the AM group exhibited blurred muscle fibers, roughly circular muscle fibers in the damaged areas, indicating severe fibrosis; the AM-Exo-miR-NC group showed a marked reduction in collagen deposition, whereas the miR-inhibitor compromised the protective effect of miR-24-3p against fibrosis. Oil Red O staining demonstrated an increase in lipid accumulation in the AM group, which was significantly reduced in the AM-Exo-miR-NC group, indicating a decrease in lipotoxicity (Figure 4D). Using ELISA, we detected significantly elevated levels of the inflammatory cytokines TNF-α and IL-6 in the serum of the Abdominal Muscle (AM) group compared to the Control group, indicative of a robust inflammatory response. In contrast, the AM-Exo-miR-NC group exhibited a marked reduction in these cytokine levels, suggesting that exosomal miR-24-3p can modulate the inflammatory response following muscle injury (Figure 4E).

Collectively, these findings demonstrate that exosomal miR-24-3p can ameliorate symptoms associated with abdominal muscle injury in mice, such as reducing muscle fiber damage, combating fibrosis, and alleviating inflammatory responses.

### 3.5 Restoration of glucose and lipid metabolism in muscle-injured mice by exosomal miR-24-3p

**FIGURE 5:**
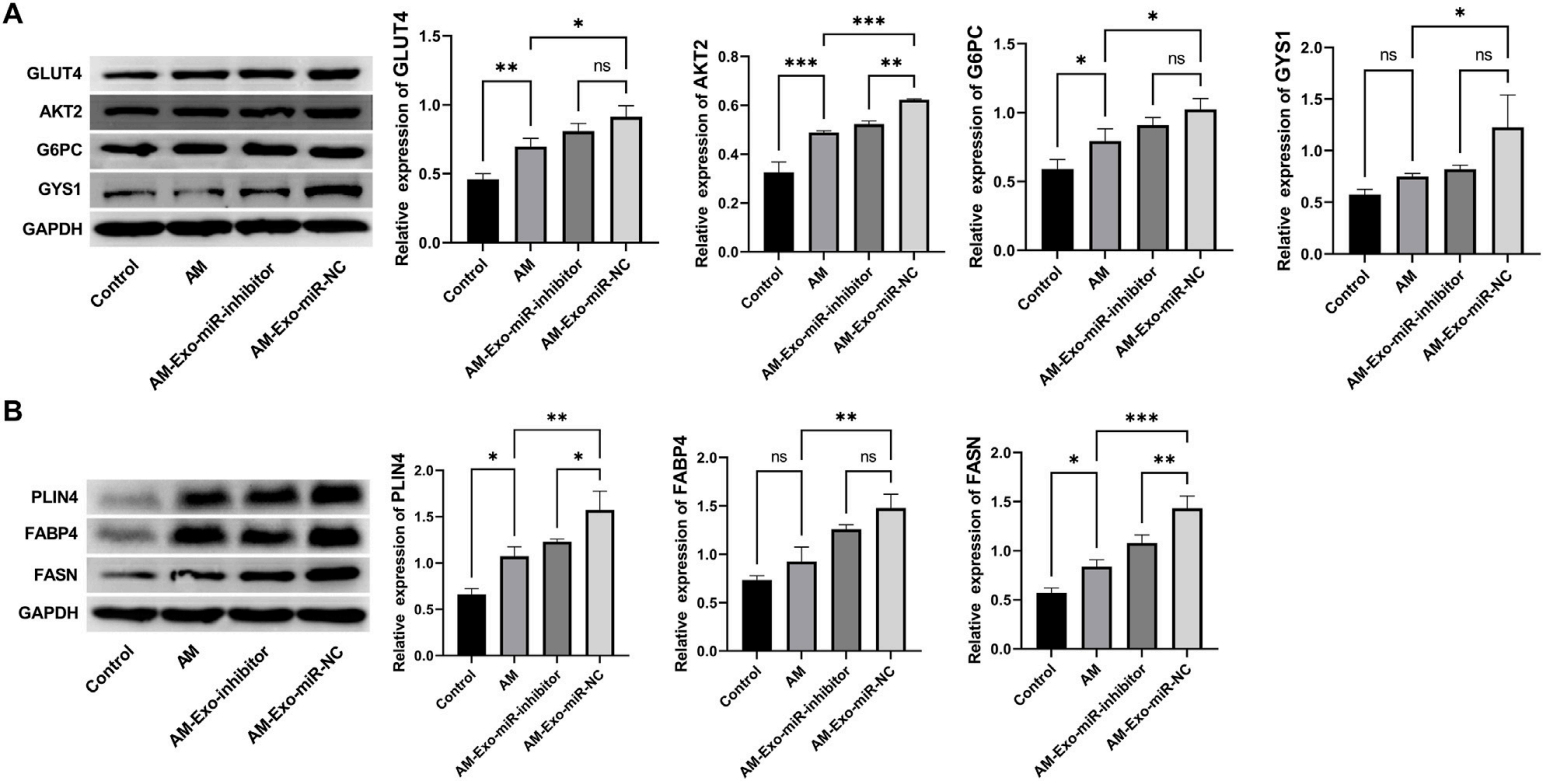
Restoration of glucose and lipid metabolism in muscle-injured mice by exosomal miR-24-3p. **(A,B)** Western blot analysis of glucose metabolism markers (GLUT4, AKT2, G6PC, GYS1) and lipid metabolism-related proteins (PLIN4, FABP4, FASN) in muscle tissues across experimental groups (AM, AM-Exo-miR-NC, AM-Exo-miR-inhibitor). GAPDH was used as a loading control. n = 3, **p < 0.01, ***p < 0.001.

In our investigation into the reparative effects of exosomal miR-24-3p on muscle injury, Western blot analysis was conducted to evaluate the expression profiles of proteins pivotal to glucose metabolism, namely GLUT4, AKT2, G6PC, and GYS1, as well as those integral to lipid metabolism, including PLIN4, FABP4, and FASN, across various experimental groups. The analysis revealed that in the Control group, the baseline expression levels of GLUT4, AKT2, G6PC, and GYS1 indicated normal glucose uptake and metabolic processes. Conversely, the Abdominal Muscle (AM) group exhibited a significant reduction in the expression of these proteins, suggesting a detrimental impact of muscle injury on glucose metabolism. Strikingly, the AM-Exo-miR-NC group demonstrated a marked restoration in the expression levels of GLUT4, AKT2, G6PC, and GYS1, while the inhibition of miR-24-3p attenuated this regulatory effect, underscoring the potential of exosomal miR-24-3p to play an essential role in the recovery of glucose metabolism post-muscle injury (Figure 5A). Regarding proteins associated with lipid metabolism, PLIN4, FABP4, and FASN displayed distinct expression patterns among the groups. Compared to the Control group, the AM group showed a significant decrease in the expression of these proteins, likely reflecting the disruption of lipid metabolism due to muscle injury. In contrast, the AM-Exo-miR-NC group exhibited an upregulation of PLIN4, FABP4, and FASN, suggesting that exosomal miR-24-3p may be involved in the resumption of lipid metabolism and potentially mitigating lipotoxicity associated with muscle injury (Figure 5B).

**FIGURE 6:**
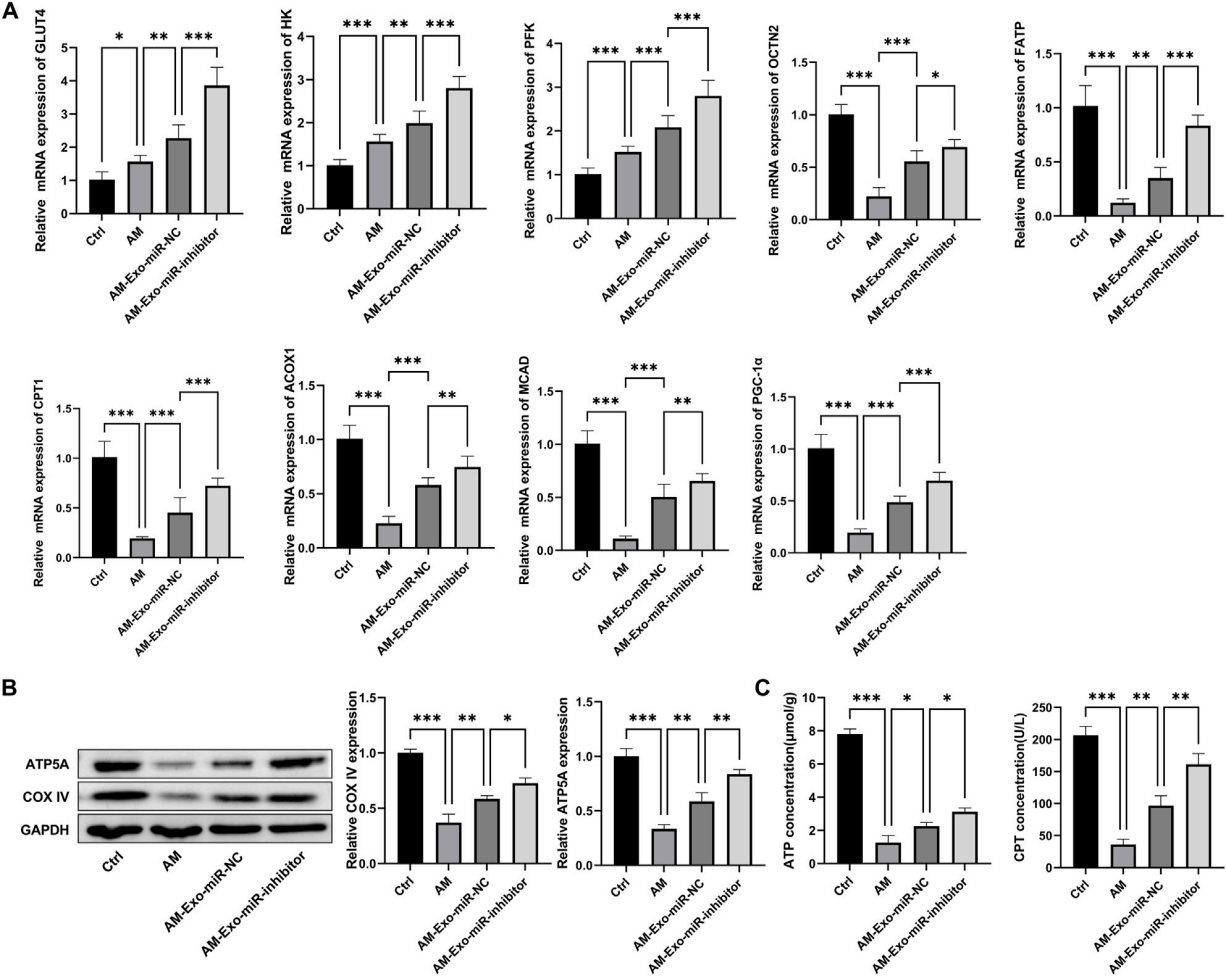
Metabolic-related experiments in muscle-injured mice. **(A)** qRT-PCR was performed to detect the mRNA expression of genes involved in fatty acid oxidation (ACOX1, CPT1, FATP) and glucose uptake (GLUT4, HK), as well as PFK and PGC-1α. **(B)** Western blot was used to analyze the protein expression of ATP5A and COX IV. **(C)** ELISA was conducted to measure ATP concentration and CPT activity. n = 3, *p < 0.05, **p < 0.01, ***p < 0.001.

To address the concern regarding the restoration of glucose and lipid metabolism, we conducted comprehensive experiments. For gene expression analysis via qRT-PCR (Figure 6A), compared with the AM group (muscle - injured mice), the AM-Exo-miR - NC group showed a significant upregulation in the expression of genes involved in fatty acid oxidation (ACOX1, CPT1, FATP) and glucose uptake (GLUT4, HK). Genes such as PFK and PGC-1α also demonstrated notable upregulation, indicating improved metabolic regulation. In contrast, when compared with the AM-Exo-miR-NC group, the AM-Exo-miR-inhibitor group exhibited a significant downregulation in these beneficial metabolic genes. Western blot results (Figure 6B) revealed that the AM-Exo-miR-NC group had increased expression of ATP5A and COX IV, key proteins for mitochondrial function, suggesting enhanced mitochondrial activity. Conversely, the AM-Exo-miR-inhibitor group showed a decrease in the expression of these proteins. ELISA analysis (Figure 6C) showed that the AM-Exo-miR-NC group had higher ATP concentrations and CPT activity, reflecting enhanced energy metabolism. In stark contrast, the AM-Exo-miR-inhibitor group presented lower ATP levels and CPT activity. These results collectively provide substantial evidence for the role of exosomal miR-24-3p in promoting the restoration of glucose and lipid metabolism in muscle - injured mice, with the inhibitor group showing the opposite trend, further validating the regulatory effect of miR-24-3p.

### 3.6 Exosomal miR-24-3p promotes muscle repair by enhancing myoblast proliferation and differentiation

**FIGURE 7:**
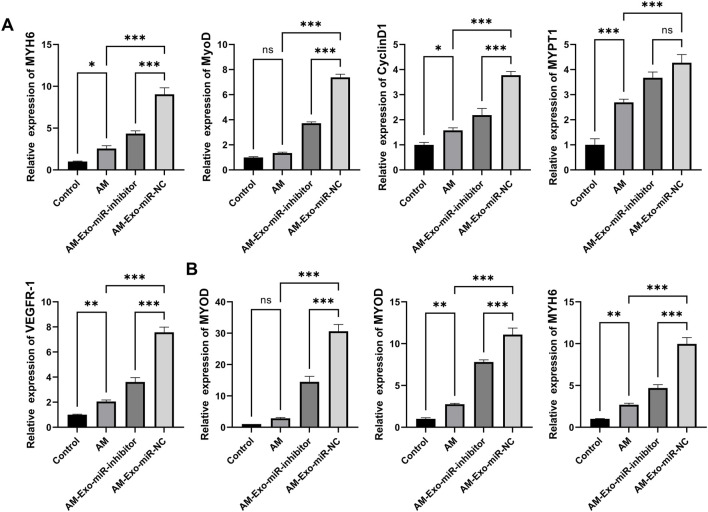
Exosomal miR-24-3p promotes muscle repair by enhancing myoblast proliferation and differentiation. **(A)** mRNA levels of MYH6, MYOD, CyclinD1, MYPT1, and VEGFR-1 were analyzed using quantitative RT-PCR. **(B)** mRNA levels of MYOD, MYOG, and MYH6 were analyzed using quantitative RT-PCR. n = 3, **p < 0.01, ***p < 0.001.

Utilizing qRT-PCR, we evaluated the impact of exosomal miR-24-3p on muscle cell proliferation and differentiation among various mouse groups. We selected the proliferation markers MYH6, MyoD, CyclinD1, MYPT1, and VEGFR-1 for our analysis. The Control group exhibited expression levels of these markers that were consistent with standard muscle physiology. In stark contrast, the Abdominal Muscle (AM) group demonstrated a significant decrease in the expression of these proliferation-related factors, indicative of impaired muscle cell proliferation as a consequence of injury. Notably, the AM-Exo-miR-NC group showed a substantial restoration in the expression levels of MYH6, MyoD, CyclinD1, MYPT1, and VEGFR-1. In comparison, the AM-Exo-miR-inhibitor group showed only a slight recovery in the expression of these proteins, which was significantly less than that observed in the AM-Exo-miR-NC group. This suggests that exosomal miR-24-3p potentially promotes muscle cell proliferation and contributes to the repair of muscle tissue (Figure 7A). In the context of myoblast differentiation, the key determinants MYOD, MYOG, and MYH6 were analyzed. Relative to the Control group, the AM group presented with diminished expression of these differentiation factors, which could jeopardize the essential differentiation processes required for muscle regeneration. Nonetheless, the AM-Exo-miR-NC group revealed an upregulation of MYOD, MYOG, and MYH6. The inhibition of miR-24-3p attenuated the beneficial effects observed in the AM-Exo-miR-NC group, suggesting that exosomal miR-24-3p has the potential to augment muscle cell differentiation and, by extension, facilitate the muscle repair cascade (Figure 7B). These findings demonstrate that exosomal miR-24-3p plays a significant role in the restoration of muscle cell proliferation and differentiation within a murine model of muscle injury, potentially facilitating the overall repair and regeneration of muscle tissue.

## 4 Discussion

In this study, we investigated the potential of exosomal miR-24-3p in facilitating the repair of abdominal muscle injuries, with a particular focus on the interaction between myoblasts and macrophages. We observed the impact of exosomal miR-24-3p on myoblast proliferation, migration, and differentiation, as well as its effects on inflammation and metabolism in mice with muscle injuries. These observations have provided a deeper understanding of the molecular basis of muscle repair. Our findings suggest that exosomal miR-24-3p may act as a crucial regulatory molecule, coordinating muscle regeneration by influencing the crosstalk between myoblasts and macrophages. The ability of exosomal miR-24-3p to modulate these processes offers new insights into the therapeutic potential of exosome-based interventions for muscle injuries.

Macrophages are highly plastic cells, capable of differentiating into the functionally distinct M1 and M2 phenotypes in response to diverse stimuli (Yu et al., 2022). Immediately following muscle injury, macrophages primarily adopt the pro-inflammatory M1 phenotype, engaging in the clearance of damaged tissue through phagocytosis of cellular debris and secretion of pro-inflammatory cytokines, which creates a favorable milieu for subsequent reparative processes (Rybalko et al., 2015). However, sustained activation of M1 macrophages poses a risk of fibrosis, highlighting the importance of transitioning towards the anti-inflammatory M2 phenotype during tissue repair (Novak et al., 2014). In the aftermath of muscle injury, M2 macrophages contribute to the resolution of inflammation by secreting an array of cytokines and growth factors, including IL-10, CCL-18, and CCL-22 (Luo et al., 2022). Evidence suggests that these anti-inflammatory M2 macrophages foster muscle regeneration by promoting angiogenesis as well as the proliferation and differentiation of muscle cells (Daneshvar et al., 2024). M2 macrophages secrete critical factors such as fibroblast growth factor and insulin-like growth factor, which are instrumental in muscle cell proliferation and differentiation (Nawaz et al., 2022). Furthermore, M2 macrophages modulate immune responses to avert muscle cell apoptosis, thereby promoting muscle regeneration (Rybalko et al., 2015). Exosomes, which mirror the properties of their parental cells, act as a crucial intercellular communication medium within the microenvironment. Their role in skeletal muscle repair and regeneration is characterized by their engagement in muscle cell proliferation, differentiation, and migration, modulation of immune functions within skeletal muscle, and the stimulation of angiogenesis (Porcu et al., 2024; Wan R. et al., 2024). Studies have demonstrated that exosomes derived from M2 macrophages can enhance the osteogenic differentiation of mesenchymal stem cells (MSCs) and modulate adipogenesis by regulating molecules such as miRNA (Li et al., 2021). Consequently, exosomes from M2-like macrophages are emerging as a promising therapeutic modality for the treatment of muscle injury.

In the present study, murine macrophage RAW264.7 cells were directed to polarize towards the M2 phenotype utilizing IL-4 and IL-10. Subsequent to the transfection-induced modulation of miR-24-3p levels within M2 macrophages, exosomes were meticulously isolated from these cells. The *in vitro* co-culture experiments, in which M2 macrophage-derived exosomal miR-24-3p was incubated with C2C12 cells, demonstrated that exosomal miR-24-3p was efficiently internalized by C2C12 cells, thereby enhancing their proliferation and migratory capabilities, as well as their involvement in cellular differentiation. Additionally, within a murine model of muscle injury, the administration of exosomal miR-24-3p was found to significantly expedite muscle repair processes. Collectively, these findings position exosomal miR-24-3p as a potentially efficacious therapeutic approach for the repair of muscle injuries. Notably, while our findings show that exosomal miR-24-3p enhances myoblast activity, previous work in diabetic wound healing reported that miR-24-3p inhibition promotes angiogenesis via targeting PIK3R3 (Xu et al., 2020). This apparent discrepancy highlights the context-dependent functions of miR-24-3p, which are influenced by cell type (e.g., myoblasts vs. endothelial cells) and disease milieu. In muscle repair, miR-24-3p likely acts through distinct targets to stimulate myogenic processes, underscoring the specificity of its role in skeletal muscle regeneration. While our experiments demonstrate specific internalization of M2-exosomes by C2C12 myoblasts, the uptake specificity across other cell types in the muscle microenvironment (e.g., resident fibroblasts, endothelial cells, or immune cells) remains to be explored. Co-culture experiments with these cell populations could reveal whether M2-exosomes exhibit preferential targeting to myoblasts or engage in broader intercellular communication, which may influence the therapeutic specificity of exosomal miR-24-3p. These studies represent an important avenue for future research to characterize the microenvironmental dynamics of exosome-mediated signaling.

Recently, some researches have implicated miR-24-3p as a potential regulatory molecule in the context of muscle injury repair. The transcription of miR-24-3p originates from two polycistronic units, namely miR-23a-27a-24-2 and miR-23b-27b-24-1, both of which generate the same mature miR-24-3p sequence (Marchetti et al., 2020). In ischemic muscle, the expression of miR-24-3p is markedly upregulated, where it plays a pivotal role in muscle repair and regeneration by modulating components of the Notch signaling pathway, including Notch-1 and Dll-1 (Marchetti et al., 2020). Moreover, miR-24-3p is engaged in the Wnt signaling pathway, which is instrumental in muscle proliferation. Dual-luciferase assays have revealed that miR-24-3p targets three shared genes within the Wnt signaling cascade—WNT4, CAMK2B, and TCF7—in both cattle and mice (Yang et al., 2022). In skeletal muscle, miR-24-3p has been shown to stimulate proliferation and impede apoptosis in C2C12 cells through the targeting of CAMK2B (Yang et al., 2022). Specifically speaking, through the transfection of miR-24-3p mimics, the expression levels of miR-24-3p were significantly elevated, leading to a marked decrease in the expression levels of cell proliferation-related genes, such as CYCLINE, PCNA, and BAX, and a marked increase in the expression levels of apoptosis-related genes, including CASPASE9, CASPASE3, and CASPEAS8. This regulatory mechanism suggests that miR-24-3p promotes cell survival and proliferation by inhibiting the expression of pro-apoptotic genes (Yang et al., 2022). miR-24-3p also suppresses the proliferation of progenitor cells sourced from fetal bovine skeletal muscle by targeting ACVR1B (Lin et al., 2022). In our experiments involving the treatment of C2C12 cells with exosomal miR-24-3p, we observed that exosomal miR-24-3p enhances the proliferative capacity of C2C12 cells, potentially through the upregulation of proliferation-associated genes such as PCNA, CyclinD1, and CDK2 (Huang K. et al., 2024). The migratory ability of C2C12 cells is crucial for muscle repair following injury, as these cells must migrate to the site of damage to facilitate the formation of new muscle fibers (Wang et al., 2024). Compared to previous studies, we have discovered that exosomal miR-24-3p promotes the migratory ability of C2C12 cells. However, the regulation of cell migration by miR-24-3p has primarily been focused on cancer cells (Yu et al., 2017), and the specific mechanisms by which miR-24-3p regulates the migration of C2C12 cells remain unclear. Additionally, future research directions may include investigations into the role of M2 macrophage-derived exosomes in promoting the migration of C2C12 cells. Although our findings establish that exosomal miR-24-3p influences myoblast proliferation and migration, the specific intracellular targets driving these processes are not fully defined. Bioinformatics analyses (TargetScan, miRDB) propose candidates like CAMK2B, Notch1, and TCF7, which are involved in myogenic differentiation and cytoskeletal regulation. These potential targets warrant validation through luciferase reporter assays and functional experiments to clarify the molecular mechanisms by which miR-24-3p modulates muscle cell behavior, a direction we plan to explore in future studies.

The C2C12 cell line, renowned for its swift differentiation into mature muscle cells, plays a pivotal role in muscle injury repair (Yang et al., 2024). Within this reparative process, C2C12 cells contribute to muscle regeneration by differentiating into multinucleated myotubes and muscle fibers, effectively replacing the damaged skeletal muscle cells (Zhang et al., 2021). The orchestration of C2C12 cell differentiation is regulated by a multitude of factors, most notably the myogenic regulatory factors (MRFs), which include myogenic factor 5 (Myf5), myogenic differentiation antigen (MyoD), myogenin (MYOG), and myogenic factor 4 (MRF4). These MRFs are integral components of the basic helix-loop-helix family of transcription factors that dictate the differentiation trajectory of skeletal muscle cells (Huang C. Y. et al., 2024; Tao et al., 2024; Wan J. et al., 2024). Recent research has highlighted the role of vitamin C in augmenting the expression and nuclear translocation of cysteine-rich protein 3 (CSRP3), which interacts with MyoD and MyoG, thereby promoting C2C12 cell differentiation and expediting the repair process in a murine model of muscle injury (Li et al., 2022). In our investigation, exosomal miR-24-3p was identified as a promoter of myoblast differentiation, achieving this by upregulating the expression of MYOD, MYOG, and MYH6.

*In vivo*, the ameliorative effects of exosomal miR-24-3p on abdominal muscle injury in mice underscore the potential of this molecule as a therapeutic agent. The observed reduction in muscle fiber damage, fibrosis, and inflammation within the treatment group may be attributed to the immunomodulatory properties of macrophage-derived exosomal miR-24-3p. Furthermore, our study elucidates the impact of exosomal miR-24-3p on the glucose and lipid metabolism in muscle-injured mice. The restoration of metabolic balance is a critical aspect of muscle repair, ensuring that the energy demands of regenerating muscle are met (Liu M. et al., 2023). Exosomal miR-24-3p may facilitate this process by modulating the expression of proteins involved in glucose and lipid metabolism, such as GLUT4 and AKT2, which are essential for glucose uptake and utilization, and PLIN4 and FABP4, which are implicated in lipid metabolism.

Our study offers significant insights into the role of exosomal miR-24-3p in muscle repair; however, it is not without its limitations, which deserve mention. Primarily, a notable limitation of this study is the lack of direct *in vitro* production and purification of exosomal miR-24-3p as a standalone therapeutic agent. To address this, future research will involve synthesizing miR-24-3p-enriched exosomes using engineered cell lines, followed by *in vivo* validation in abdominal muscle injury models. This approach will directly demonstrate the specificity of exosomal miR-24-3p in promoting muscle repair and establish its potential as a cell-free therapeutic candidate. Our investigation centers on the *in vitro* impact of exosomal miR-24-3p on C2C12 cells and its *in vivo* effects within a murine model, which may not entirely capture the intricacies of human muscle physiology. Furthermore, the precise intracellular pathways mediating the effects of exosomal miR-24-3p on myoblast migration and differentiation are not fully delineated and require additional investigation. The long-term impact of exosomal miR-24-3p on muscle functionality and any potential off-target effects were also beyond the scope of this study. Future endeavors should endeavor to bridge these knowledge gaps by expanding the scope to include human cell studies and by undertaking long-term studies to comprehensively assess the therapeutic potential and safety of exosomal miR-24-3p in the context of muscle repair. To further dissect the cellular and molecular mechanisms of exosomal miR-24-3p, future studies could employ advanced technologies, such as spatial CITE-seq for high-plex protein and transcriptome co-mapping at cellular resolution (Liu Y. et al., 2023), perturb-DBiT for spatially resolved *in vivo* CRISPR screen sequencing to identify novel target genes (Baysoy et al., 2024), and multimodal tri-omics mapping to analyze spatial dynamics of macrophage-myoblast interactions during muscle regeneration (Fan et al., 2024). These techniques would provide unprecedented insights into the spatial and functional heterogeneity of the repair microenvironment, bridging mechanistic discoveries with translational applications.

## 5 Conclusion

In conclusion, our study demonstrates that exosomal miR-24-3p plays a significant role in facilitating abdominal muscle repair by modulating myoblast-macrophage crosstalk, highlighting its potential as a therapeutic target for muscle injuries.

## Data Availability

The raw data supporting the conclusions of this article will be made available by the authors, without undue reservation.
